# Counterfactual Reasoning Deficits in Schizophrenia Patients

**DOI:** 10.1371/journal.pone.0148440

**Published:** 2016-02-01

**Authors:** Fernando Contreras, Auria Albacete, Pere Castellví, Agnès Caño, Bessy Benejam, José Manuel Menchón

**Affiliations:** 1 Psychiatry Department, Bellvitge University Hospital-IDIBELL, Barcelona, Spain; 2 Department of Clinical Sciences, School of Medicine, University of Barcelona, Barcelona, Spain; 3 Carlos III Health Institute, Centro de Investigación Biomédica en Red de Salud Mental (CIBERSAM), Barcelona, Spain; 4 Department of Psychology, University of Girona, Girona, Spain; Benito Menni Complejo Asistencial en Salud Mental, SPAIN

## Abstract

**Background:**

Counterfactual thinking is a specific type of conditional reasoning that enables the generation of mental simulations of alternatives to past factual events. Although it has been broadly studied in the general population, research on schizophrenia is still scarce. The aim of the current study was to further examine counterfactual reasoning in this illness.

**Methods:**

Forty schizophrenia patients and 40 controls completed a series of tests that assessed the influence of the “causal order effect” on counterfactual thinking, and the ability to generate counterfactual thoughts and counterfactually derive inferences from a hypothetical situation. Socio-demographic and clinical characteristics, as well as neurocognitive variables, were also examined.

**Results:**

Compared to controls, the schizophrenia patients generated fewer counterfactual thoughts when faced with a simulated scenario. The pattern of response when assessing the causality effect of the order was also different between the groups, with the patients being more frequently unable to attribute any ordering of events than the control subjects. Additionally, the schizophrenia patients showed more difficulties when deriving normative counterfactual inferences from hypothetical social situations. None of the counterfactual reasoning measures was associated to any of the cognitive functions or clinical and socio-demographic variables assessed.

**Conclusions:**

A global impairment in counterfactual thinking characterizes schizophrenia patients. Because of the potential impact of such deficits on psychosocial functioning, targeting counterfactual reasoning for improvement might be considered in future treatment approaches.

## Introduction

Counterfactual thinking (CFT) is a specific type of conditional reasoning that takes place when thinking about past events. In this context, most people automatically compare the actual outcome of the event with “what might have been” by generating hypothetical “if only” outcomes supposing an alternative event had taken place [1,2]. For instance, in the fictional situation where John has failed an important test, he might generate a counterfactual thought like, *If I had studied more*, *I could have passed the test*. Theoretically, CFT has been framed in *norm theory* [3] as a biased decision-making process, and in the *mental models perspective* [4] as one of the “building blocks of reasoning.” Used in response to real-world experiences, counterfactual reasoning relies on mental models of alternative possibilities that are represented in the form of mental simulations [5]. There is general agreement that CFT is related to other processes such as problem-solving [1], causal judgements [6] and deductive reasoning [7], as well as being important for mood regulation [6] and having a daily life coordination function, influencing behavioural changes and performance improvement [8].

CFT has been studied by examining the ability of individuals to generate counterfactual alternatives, as well as by looking at other effects that have been considered to influence CFT such as order effects. One of the most studied of these latter effects is the “causal order effect,” which describes how, when faced with a hypothetical scenario involving a chain of events that has a negative outcome, most subjects tend to choose the first event in the scenario as the main determinant of the outcome [9]. CFT processes have also been studied by examining inferences resulting from CFT in the face of hypothetical social events [3,10].

Regarding CFT’s neuroanatomical correlates, the involvement of the prefrontal cortex (PFC) generally and, more specifically, the orbitofrontal cortex (OFC) has been evidenced by studies of patients with PFC lesions [11], traumatic injury to the frontal lobe [12], Parkinson’s dementia [13] and Huntington’s disease [14]. Patients with these disorders demonstrate difficulties in generating a normal level of counterfactual alternatives as well as in foreseeing the possible negative consequences of their own actions. This may contribute to their tendency to persevere with certain behaviours and strategies that have been proven no longer beneficial. Functional neuroimaging studies have also found that the PFC is activated during CFT tasks [15,16], and more specifically that the OFC is activated in decision-making that has CFT components [17].

Schizophrenia is, among its other clinical features, characterized by delusions and disturbances in the logical structure of thought. Additionally, the disorder is linked to prefrontal dysfunction [18,19], and patients show impoverished decision-making and problem-solving skills, logical reasoning alterations [20], as well as a tendency to perseverate and to have a poor ability to generate novel ideas and plan for the future [21,22]. The presence of cognitive biases involved in the formation and maintenance of positive symptoms has also been increasingly recognized in recent years [23–25]. These biases include an information-gathering cognitive style that is characterized by jumping to conclusions, externalizing attributional biases, and deficits in understanding social situations and the intentions of others—Theory of Mind (ToM) deficits [26]. It is not clear, though, whether these biases are independent from each other or whether, on the contrary, they represent parts of a yet undetected whole [27]. Taking into account that, in healthy people, CFT is known to play a role in the false belief reasoning development [28], and to enhance memory distortions (e.g., hindsight bias) that contribute to suboptimal decision-making [29], but also to be involved in ToM deficits in schizophrenia patients (i.e., difficulties in the processing of counterfactual information such as sarcasm) [30], it would be interesting to explore this type of conditional thinking within the cognitive biases tradition. Also relevant is the impact these cognitive deficiencies might have on schizophrenia patients’ personal and social functioning—on the difficulties they present in their everyday activities, interpersonal relationships, or academic and work performance [31].

However, studies on the relationship between schizophrenia and CFT are scarce, even though this type of investigation involves an interesting and innovative application of a paradigm from experimental cognitive psychology. To our knowledge, there has been only one study to date, which was carried out on a relatively small sample of 14 schizophrenia patients and 12 healthy controls and which used two different CFT measures. First, the generation of spontaneous counterfactual alternatives was explored by asking the participants to recall a negative personal event, after which the total number of thoughts about how this event could have turned out differently was recorded. Second, the ability to make counterfactually derived inferences was assessed using a measure specifically designed for the study, the Counterfactual Inference Test (CIT). The results indicated that the patients generated fewer counterfactual thoughts than the controls and showed a different pattern of responses when counterfactually deriving inferences. Both impairments were related to the patients’ deterioration in social functioning but not to cognitive measures including the Vocabulary and Digit Span subtests of the Wechsler Adult Intelligence Scale (WAIS-R) and with the FAS Verbal Fluency test [32].

The aim of the present study was to extend previous research examining CFT in schizophrenia, using a larger sample of patients and control subjects. Moreover, to our knowledge, this is the first time that the causal order effect in CFT has been employed in this patient group. The study further examined whether CFT performance was related to any basic cognitive domains using a detailed neuropsychological battery of tests designed to assess cognitive impairment in schizophrenia—the Brief Assessment of Cognition in Schizophrenia (BACS) [33,34]. Potential associations with socio-demographic and clinical variables were also explored.

## Materials and Methods

### Study design

This case-control study was conducted in the outpatient services of the Psychiatry Department of Bellvitge University Hospital in Hospitalet de Llobregat, Barcelona, Spain. The Clinical Research Ethics Committee of Bellvitge (CEIC) approved all study procedures, and all subjects gave written informed consent before inclusion.

### Participants

Forty schizophrenia patients who met DSM-IV-TR [35] criteria were included in the study. Subjects with diagnoses of bipolar, schizoaffective, delusional or other Axis I disorders were excluded. Four of the schizophrenia patients had been treated with electroconvulsive therapy at least once in their lives, but not within the six months prior to entering the study. Forty healthy control subjects without a history of personal or family psychiatric illness or substance use disorder were recruited from hospital employees. All participants were excluded if they had a history of brain injury, an estimated Intelligence Quotient (IQ) lower than 70 or a mental disease due to a medical condition. The groups were matched for gender, age and educational level.

### Measures and procedures

Mental and personality disorders were assessed in both groups using the structured clinical interview for DSM-IV Axis I Disorders (SCID-I) [36] and Axis II Personality Disorders (SCID-II) [37] prior to enrolment. The examination of the schizophrenia patients was performed on two consecutive days: on the first day, clinical variables were recorded and cognitive function was assessed; on the second day, CFT was evaluated. The assessment of the healthy controls was carried out in a single session. Socio-demographic and clinical variables were recorded by means of an in-house standardized evaluation. Symptoms and severity of illness were assessed using the Spanish version of the Positive and Negative Syndrome Scale (PANSS) [38,39] and the Clinical Global Impression-Schizophrenia Scale (CGI-SCH) [40]. Level of functioning was assessed using the Global Assessment of Functioning (GAF) scale [41]. Pharmacological treatment was recorded, and daily dose equivalents of chlorpromazine were calculated [42].

#### Neuropsychological testing

A broad range of cognitive domains were evaluated with the Brief Assessment of Cognition in Schizophrenia (BACS), in the Spanish validation of which our group participated [33]. The functions assessed were verbal memory, working memory, motor function, verbal fluency, attention and processing speed, and executive function. Finally, the Spanish version of the vocabulary subtest of the Wechsler Adult Intelligence Scale battery [43] was administered to give an estimate IQ that was relatively resistant to postmorbid decline in the patients.

#### CFT evaluation

CFT was evaluated with three different tests given in the following order: assessment of the causal order effect, generation of counterfactual thoughts and the ability to make counterfactually derived inferences.

The first two measures were assessed through the research paradigm proposed originally by Wells et al. [9], which consists of a written scenario of four consecutive independent events that result in a negative outcome. In order to avoid first event bias, the researcher randomly changed the order of the events using a 4x4 Latin square design. All participants had to read the scenario, which in brief consisted of an individual who heard on the radio that a store on the other side of town had a great sale on a limited number of stereo systems. His/her progress in getting to the store was impeded by four consecutive minor misfortunes: a) a speeding ticket, b) a flat tire, c) a traffic jam, and d) a group of elderly people crossing the street. Because of these mishaps, he/she arrived late only to find out that the last stereo system had already been sold just a few minutes earlier. This scenario provided the basis for two experiments that were carried out.

First of all, in *Experiment 1*, designed to assess the causal order effect, participants were asked to choose and justify which of the four events was, in their opinion, the most probable cause for the negative outcome and, therefore, the event that they would select in order to undo the scenario. Participants who, even when encouraged, were still unable to choose one of the events, were directly assigned the response type “reasoning blocking.” This was done to ensure that these responses were not considered as missing data. The time given to participants to complete this experiment was 60 seconds. Researchers recorded each participant’s answer.

Secondly, in *Experiment 2*, the generation of counterfactual thoughts was evaluated by asking the participants to write down possible alternative ways they could have arrived in time to buy the stereo system; these could be either new original alternatives (e.g., “If only I had called and made a reservation in advance”) or alternatives that changed one of the “unfortunate” events (e.g., “If only I hadn’t been speeding”). Participants were given five minutes to complete this experiment. The number of different counterfactual thoughts produced was recorded by two independent researchers, who filtered which of the participants’ answers were real CFT answers and which ones were illogical or bizarre answers (e.g., “I continued sleeping”).

Finally, the Counterfactual Inference Test (CIT), originally developed by Hooker et al. [32], was administered to measure ability to generate counterfactually derived inferences. This test is based on previous research which has shown that CFT influences affective and judgemental (cognitive) reactions regarding social events, and also that CFT is heightened in the face of outcomes preceded by unusual rather than typical actions [3], as well as when individuals are faced with events that seem “almost” (either spatially or temporally) to have occurred [10]. The CIT (for an overview of the test, see Table 1) consists of a set of four forced-choice questions; for each, two events with similar outcomes experienced by two subjects are presented. However, the circumstances between them differ such that in one the subjects should think “if only” to a greater extent than in the other. The target questions vary to reflect different higher order inferences. Therefore, item 1 focuses on a general affective reaction (“upset”) in the context of a spatial “nearly happened” event; item 2 on a general affective reaction (“regret”) in response to an “unusual” event; item 3 on a judgemental or cognitive reaction (“rumination”) brought on by a temporal “nearly happened” event; and item 4 on a judgemental or cognitive reaction (“judgements of avoidance/prevention”) in the face of an “unusual” event [32]. Each of the four questions describes a hypothetical social event and participants are given three possible answers: a normative answer (that is, the target counterfactual response), a non-normative response and a “same/can’t tell” response if the participant considers none of the previous options to be suitable. The CIT total score is calculated from the typical/normative pattern of responses, based on previous research using a sample of undergraduate control subjects [32]. Each item on the test is given a score of 1 if the subject chooses the normative answer—that is, the option where the subject would most probably think “if only”; if the subject chooses any of the other answers (non-normative or “same/can’t tell”) they receive a score of zero. Therefore, the total score may range between 0 and 4, with greater values indicating a counterfactual response closer to a normative pattern. Participants were given five minutes to complete the test.

**Table 1:** Counterfactual Inference Test (CIT) [32].

Items	Response
ITEM 1: Reaction of upset (affective) in response to a spatial “nearly happened” event.	**a) Janet**
*Janet is attacked by a mugger only 10 feet from her house*. *Susan is attacked by a mugger a mile from her house*. *Who is more upset by the mugging*?	b) Susan
	c) Same/Can’t tell
ITEM 2: Reaction of regret (affective) in response to an “unusual” event.	a) Ann
*Ann gets sick after eating at a restaurant she often visits*. *Sarah gets sick after eating at a restaurant she has never visited before*. *Who regrets their choice of restaurant more*?	**b) Sarah**
	c) Same/Can’t tell
ITEM 3: Reaction of rumination (judgemental) in response to a temporal “nearly happened” event.	a) Ed
*Jack misses his train by five minutes*. *Ed misses his train by more than an hour*. *Who spends more time thinking about the missed train*?	**b) Jack**
	c) Same/Can’t tell
ITEM 4: Reaction of avoidance (judgemental) in response to an “unusual” event.	**a) Bob**
*John gets into a car accident while driving on his usual way home*. *Bob gets into a car accident while trying a new way home*. *Who thinks more about how his accident could have been avoided*?	b) John
	c) Same/Can’t tell

*Note*. The typical/normative pattern of responses are indicated in boldface [32].

### Statistical analysis

For the descriptive analyses, absolute and relative frequencies were calculated for categorical variables. Continuous variables were assessed using the mean and standard deviation (SD) for normally distributed variables and the median and interquartile range (Q1-Q3) for non-normally distributed variables. In order to detect differences between groups, Fisher’s exact test and χ^2^ were used for categorical data, whereas the t-test and the Mann-Whitney-Wilcoxon test were applied to parametric and non-parametric continuous data, respectively. Normality of distributions was checked using the Kolmogorov-Smirnov test. In Experiment 1, in order to test whether the observed proportions departed from the expected p = 0.25 (i.e., equally distributed frequencies per category in each group was considered the null hypothesis), a χ^2^ goodness of fit test was performed on each group. In multivariate analyses, linear and logistic regression models were used depending on whether the dependent variable was considered continuous or binary respectively. Finally, General Linear Models were used to assess the possible influence of cognitive variables on CFT measures, generating models adjusted for gender, age and educational level. In all analyses, the differences were assessed using a statistical test based on two-tailed significance at p = 0.05. To account for the multiple comparison issue, the False Discovery Rate (FDR) suggested by Benjamini and Hochberg was applied [44]. The data were managed and analyzed using the statistical software package SPSS (Version 18.0 for Windows SPSS, Inc., Chicago, Ill).

## Results

### Socio-demographic and clinical characteristics, and neuropsychological performance

Socio-demographic characteristics and neurocognitive measures are summarized in Table 2. More schizophrenia patients were unemployed and single at enrolment, and they obtained statistically significantly lower scores in all cognitive domains than the healthy control subjects. Clinical characteristics for the patients group are shown in Table 3.

**Table 2:** Socio-demographic characteristics and neurocognitive measures.

	Schizophrenia Patients (n = 40)	Healthy Controls (n = 40)	p-value
**Socio-demographic characteristics**			
Male gender, *n (%)*	23 (57.5)	25 (62.5)	0.65
Age (years)	39.4 (12.2)	39.8 (12.3)	0.88
Educational level (years)	10.4 (3.6)	11.2 (3.3)	0.27
Employment status, *n (%)*			<0.001
Employed	13 (32.5)	35 (87.5)	
Student	2 (5.0)	0 (0.0)	
Unemployed/Retired	25 (62.5)	5 (12.5)	
Civil status, *n (%)*			0.02
Married	7 (17.5)	17 (42.5)	
Single	32 (80.0)	20 (50.0)	
Divorced	1 (2.5)	3 (7.5)	
Hand Dominance (right /left), *%*	95.0/5.0	97.5/2.5	0.56
**Neurocognitive measures**			
Estimated Intelligence Quotient	97.3 (12.2)	111.6 (8.9)	<0.001
Verbal memory	34.0 (12.0)	43.7 (6.5)	<0.001
Working memory	14.6 (4.2)	18.4 (3.3)	<0.001
Motor function	67.5 (16.6)	82.9 (9.2)	<0.001
Verbal fluency	27.8 (10.3)	44.7 (8.6)	<0.001
Processing speed	35.4 (14.6)	53.7 (8.4)	<0.001
Executive function	17.9 (3.0)	19.1 (1.7)	0.039

*Note*. Values presented as means (standard deviation) unless specified otherwise.

**Table 3:** Clinical measures in schizophrenia patients.

Clinical measures in schizophrenia patients	
Age of onset of schizophrenia (years), *median (range)*	21.5 (15–34)
Duration of illness (years)	16.7 (10.8)
Readmissions (episodes), *median (range)*	2.0 (0–12)
Suicide attempts (episodes), *median (range)*	0.00 (0–4)
CGI-SCH	14.88 (3.1)
GAF, *median (range)*	60.0 (50–80)
Pharmacological treatment^a^	548 (373)
PANSS Dimensions	
Positive symptoms	13.5 (3.4)
Negative symptoms	22.1 (5.9)
General Psychopathology	37.6 (8.8)
Total score	73.15 (16.14)

*Note*. Values presented as means (standard deviation) unless specified otherwise. CGI-SCH: Clinical Global Impression Scale-Schizophrenia Scale; GAF: Global Assessment of Functioning; PANSS: Positive and Negative Syndrome Scale.

^a^Milligrams per day in chlorpromazine equivalents.

### CFT evaluation

#### Experiment 1: The causal order effect

Although the pattern of ordering the events to undo scenarios was found to be different between the schizophrenia patients and the healthy controls (p = 0.033; Table 4), both groups chose the first event to undo the scenario more often than the second, third or fourth event (p<0.05 in both groups). The proportion of schizophrenia patients choosing the first event was lower (45% for patients vs. 60% for control subjects), although the difference was not statistically significant (p = 0.179). The patients were significantly more frequently unable to attribute any ordering of events; in other words, they were more frequently unable to choose any event at all (22.5% vs. 5.0%; p = 0.023).

**Table 4:** Experiment 1: Descriptive and comparative analysis of the causal order effect.

	Schizophrenia Patients (n = 40)	Healthy Controls (n = 40)	p-value
**Experiment 1: The causal order effect**			
Order of the events, *n (%)*			0.033
1^st^	18 (45.0)	24 (60.0)	
2^nd^	7 (17.5)	4 (10.0)	
3^rd^	4 (10.0)	2 (5.0)	
4^th^	2 (5.0)	8 (20.0)	
Reasoning blocking^a^	9 (22.5)	2 (5.0)	0.023
1^st^ vs. 2^nd^, 3^rd^, 4^th^, reasoning blocking	18 (45.0)	24 (60.0)	0.179

^a^Unable to choose any event.

#### Experiment 2: Generation of counterfactual thoughts

The total number of answers generated spontaneously (both real and non-real counterfactual thoughts) was not significantly different between the schizophrenia patients and the healthy controls (p = 0.173). However, when only the total number of real counterfactual thoughts was taken into account, the patients generated fewer thoughts than the control group (p = 0.015; Table 5). Moreover, the proportion of subjects unable to generate any thought (that is, zero answers) was significantly higher among the schizophrenia patients than the healthy controls (22.5% vs. 5.0%). There were no statistically significant correlations regarding any measure of the study and the daily antipsychotic dose taken.

**Table 5:** Experiment 2: Descriptive and comparative analysis of the counterfactual thoughts generation.

	Schizophrenia Patients (n = 40)	Healthy Controls (n = 40)	p-value
**Experiment 2: Generation of counterfactual thoughts**			
Total number of answers generated,^a^ median (Q1*-Q3**)	2.0 (2.0–3.0)	3.0 (2.0–3.0)	0.173
Number of counterfactual thoughts, *median (Q1-Q3)*	2.0 (1.0–2.0)	2.0 (1.0–3.0)	0.015
Number of counterfactual thoughts, *n (%)*			
0	9 (22.5)	2 (5.0)	
1	10 (25.0)	9 (22.5)	
2	14 (35.0)	15 (37.5)	
3	7 (17.5)	12 (30.0)	
4	0 (0.0)	2 (5.0)	

^a^Including both real and non-real counterfactual thoughts.

*Q1: percentile 25;

**Q3: percentile 75.

#### Generation of counterfactually derived inferences

Significant differences were found for the items related to “regret” (general affective reaction) in response to an “unusual” event and to “rumination” (judgemental/cognitive reaction) in response to a temporal “nearly happened” event (Table 6). Regarding the “regret” item, a significant proportion of patients were unable to choose between the normative and the non-normative response, choosing the “same/can’t tell” answer (p = 0.042; item 2). In the case of the “rumination” item, a higher proportion of schizophrenia patients selected the non-normative response (p = 0.037; item 3) rather than choosing the normative response or the “same/can’t tell” answer.

**Table 6:** CIT: Descriptive and comparative analysis of the counterfactually derived inferences assessment.

	Schizophrenia Patients (n = 40)	Healthy Controls (n = 40)	p-value
**Total score,** median (Q1*-Q3**)	2.0 (1.3–3.0)	3.0 (2.0–3.0)	0.130
**Upset (item 1),** *n (%)*			0.415
Normative response	14 (35.0)	13 (32.5)	
Non-normative response	10 (25.0)	6 (15.0)	
Same/can’t tell	16 (40.0)	21 (52.5)	
**Regret (item 2),** *n (%)*			0.042
Normative response	21 (52.5)	31 (77.5)	
Non-normative response	11 (27.5)	7 (17.5)	
Same/can’t tell	8 (20.0)	2 (5.0)	
**Rumination (item 3),** *n (%)*			0.037
Normative response	26 (65.0)	35 (87.5)	
Non-normative response	11 (27.5)	3 (7.5)	
Same/can’t tell	3 (7.5)	2 (5.0)	
**Judgements of avoidance (item 4),** *n (%)*			0.372
Normative response	25 (62.5)	23 (57.5)	
Non-normative response	6 (15.0)	11 (27.5)	
Same/can’t tell	9 (22.5)	6 (15.0)	

*Q1: percentile 25;

**Q3: percentile 75.

However, the difference in CIT total score did not reach statistically significant differences (p = 0.130)—that is, both groups generally tended to choose the target counterfactual response.

#### CFT and socio-demographic, clinical and neurocognitive measures in the schizophrenia patients

After FDR correction, no statistically significantly associations were found between any of the CFT measures and any of the clinical, socio-demographic (S1 Table) or cognitive variables assessed (data not shown).

## Discussion

The present study focused on the assessment of CFT in patients with schizophrenia. Only one previous study has assessed CFT in this group of patients [32], and it found that they generated fewer counterfactual thoughts and showed an altered different pattern of responding compared to healthy controls. Our study examined the influence of the causal order effect in CFT, and the ability to generate counterfactual thoughts and to counterfactually derive inferences. Our results demonstrate significant alterations in schizophrenia patients on all three measures compared to well-matched controls. No significant associations were found with clinical and socio-demographic status, as well as with neuropsychological functioning.

The analyses revealed the causality attribution pattern to be significantly different between the schizophrenia patients and the healthy controls. This might be due to the fact that the patients tended to get blocked more frequently when asked to determine the event they would select in order to undo the scenario and avoid the negative outcome. In other words, and in contrast to previous research with general population, our results suggest that in schizophrenia there is a tendency to deviate from the normative ordering pattern by choosing the first event less frequently than the controls. Thus, this alteration might influence these patients’ daily functioning since they do not attribute causality in the same way. Hence, it would be interesting to study this topic in relation to conceptual disorganization or formal thought disorder in schizophrenia.

Our findings are also in line with those of a previous study that found impoverished CFT generation in schizophrenia patients [32]. In this, the patients generated less CFT alternatives when faced with simulated scenarios, but more importantly, they also tended to be unable to generate any CFT (zero answers) that would avert the negative outcome more often than healthy controls. Interestingly, though, the results also suggested that the patients that could activate CFT (i.e., they could generate at least one alternative) to a degree similar to the healthy controls. Nevertheless, it seems from both this and our study that schizophrenia is a disorder where patients experience difficulties in activating alternative representations to reality and have difficulties in re-imagining a negative outcome in a positive way using conditional reasoning.

When exploring a higher cognitive level of information processing (i.e., the generation of counterfactually derived inferences) in our “bottom-up” experimental design, we obtained results suggesting that schizophrenia patients do not follow the normative counterfactual reasoning pattern: they less frequently selected a regretful reaction in response to an “unusual” event or a judgement-related reaction in response to a “nearly happened” event. Among emotions related to CFT, the experience of regret may have an adaptive function because it can guide future decisions, based on information gathered from the outcome of previous choices [45,46]. Whereas in the normal population, most people react with greater regret to an unusual event, the reaction of the schizophrenia patients in our study was the same regardless of whether the event was usual or unusual. These findings appear to be in line with those of a previous study which found that both schizophrenia patients with prominent positive symptoms and patients with OFC lesions did not report regret and did not anticipate negative consequences resulting from their choices [47].

Regarding cognitive judgement-related reactions, the present study found that schizophrenia patients showed a lower tendency to react with rumination when faced with a negative temporal “nearly happened” event. Their worse performance here reflected the fact that they tended to disregard the negative outcome of a social event and hence exhibit a maladaptive response. These results could be considered consonant with the fact that the negative symptoms of schizophrenia (e.g., blunted affect, emotional withdrawal and apathetic social withdrawal) lead to an inability to deal with emotions or interpersonal relationships. In addition, our results might also contribute knowledge to the study of cognitive biases in schizophrenia. The different patterns of responses that our patients presented when making causality attributions and when counterfactually deriving inferences might be conceptually linked to the study of jumping to conclusions and externalizing attributional biases (e.g., choosing one event from the sequence in the causal order effect experiment) that have been demonstrated in the disorder [23–25], as well as ToM deficits [26] (e.g., perceiving the beliefs and intentions of the CIT characters).

In contradiction with previous research findings [32], our results suggest that the CFT impairment observed was not related to psychosocial functioning deficits in this sample of schizophrenia patients. However, taking in account that in the general population CFT has been proposed as a cognitive process that contributes to effective psychosocial function [8], future studies using other measures of social dysfunction in larger samples of patients may be warranted.

Furthermore, taking into account that schizophrenia is associated with compromise in almost all cognitive domains [48–50], we explored the potential link between the various cognitive functions assessed and CFT performance. However, although the neuropsychological exploration was more extensive than in previous research, the results were that none of the variables examined was associated with CFT impairment. These results are similar to those of Hooker et al. [32], who also failed to find a relationship between CFT performance and any of the neuropsychological variables they assessed. Accordingly, they proposed that CFT could not be explained either by a generalized cognitive deficit or a specific function like verbal fluency. The relationship between basic cognition and higher cognitive processes is controversial: for example, whether social cognitive and basic cognitive processes are associated is a question not yet adequately answered [51–53]. Authorities in this field like Green et al. [54] have suggested that these two domains must overlap, and the argument is about the degree to which they overlap. In the same way, debate about whether CFT deficits could be the result of a pervasive cognitive impairment or is dependent on a specific deficit in a certain cognitive domain can still be considered to be open.

The present study has some shortcomings that should be acknowledged. Firstly, it is important to note that while the sample used in this study was larger than the one used in previous research [32], it still included only a relatively small number of participants. This could have resulted in a lack of statistical power and greater chances of making a type II error, increasing the possibility that the study was not able to detect actual differences between groups. Secondly, our sample did not meet criteria for clinical stability, although the median total PANSS score was 73.1 (SD = 16.14) which indicates a relatively low level of current symptoms. Future research might consider recruiting patients in remission as defined by Andreasen et al. [55] to assure that their cognitive functions are not biased by active symptomatology. Thirdly, use of a case-control design prevents drawing conclusions on whether the CFT impairment observed originated after or before the onset of the illness.

In conclusion, findings from the current study evidence a global impairment in counterfactual reasoning of schizophrenia patients compared with healthy controls. Because of the potential ecological impact that counterfactual thinking deficits might have on these patients’ functional outcomes, we suggest that these deficiencies could be considered as a future target for treatment in schizophrenia. Finally, it would be interesting to study whether CFT could be considered a new cognitive endophenotype for schizophrenia by conducting research among healthy relatives. Considering the NIMH Research Domain Criteria project’s new approach to research, we suggest that the study of CFT might be included as a subconstruct alongside other cognitive processes [56].

## Supporting Information

S1 Table Socio-demographic and clinical measures related to CFT measures in the schizophrenia patients group (n = 40). *Note*. All p-values are adjusted by False Discovery Rate (FDR). ^a^Causal order effect assessment - 1^st^ vs. 2^nd^, 3^rd^, 4^th^, reasoning blocking. *Logistic regression; **Linear regression.
(PDF)
