# Supplementary material for: Counterfactual Reasoning Deficits in Schizophrenia Patients
Source: PLoS One. 2016 Feb 1;11(2):e0148440. doi: 10.1371/journal.pone.0148440 (PMC4734710; doi:10.1371/journal.pone.0148440)
Supplement: S1 Table — Note. All p-values are adjusted by False Discovery Rate (FDR). aCausal order effect assessment - 1st vs. 2nd, 3rd, 4th, reasoning blocking. *Logistic regression; **Linear regression. (PDF) [file pone.0148440.s001.pdf]

S1 Table. Socio-demographic and clinical measures related to CFT measures in the schizophrenia patients group (n=40).

	**Causal order effect (Experiment 1)[a]**		**Number of counterfactual thoughts generated (Experiment 2)**		**CIT Total score**	
	OR (95%CI)*	p-value*	β (95%CI)**	p-value**	β (95%CI)**	p-value**
Employment status						
Employed/Student vs. Unemployed/Retired	2.67 (0.72 to 9.95)	0.792	-0.63 (-1.29 to 0.04)	1.000	-1.04 (-1.69 to 0.40)	0.099
Civil status						
Married vs. Single/Divorced	1.11 (0.21 to 5.76)	1.000	0.46 (-0.41 to 1.33)	1.000	-0.70 (-1.61 to 0.21)	0.838
Onset of schizophrenia. years	0.97 (0.86 to 1.09)	0.934	0.02 (-0.05 to 0.08)	0.963	-0.02 (-0.08 to 0.05)	0.971
Readmissions. episodes	0.99 (0.75 to 1.31)	0.995	-0.04 (-0.19 to 0.11)	1.000	-0.02 (-0.18 to 0.14)	0.989
Suicide attempts. episodes	0.90 (0.48 to 1.68)	0.932	-0.01 (-0.34 to 0.32)	0.996	0.15 (-0.19 to 0.50)	1.000
PANSS dimensions						
Positive	0.96 (0.79 to 1.16)	0.953	-0.02 (-0.12 to 0.08)	0.987	0.01 (-0.09 to 0.12)	0.970
Negative	0.91 (0.81 to 1.02)	0.949	-0.05 (-0.10 to 0.01)	1.000	0.02 (-0.04 to 0.08)	1.000
General psychopathology	0.96 (0.89 to 1.04)	1.000	-0.01 (-0.05 to 0.02)	1.000	0.01 (-0.03 to 0.05)	0.981
Total	0.96 (0.94 to 1.02)	0.953	-0.01 (-0.03 to 0.01)	1.000	0.01 (-0.02 to 0.03)	1.000
GAF	0.98 (0.88 to 1.09)	0.949	-0.02 (-0.08 to 0.04)	1.000	0.00 (-0.06 to 0.06)	1.000
CGI-SCH	0.93 (0.76 to 1.14)	1.000	0.00 (-0.11 to 0.11)	0.985	0.04 (-0.07 to 0.16)	1.000

Note. All p-values are adjusted by False Discovery Rate (FDR).
[a]Causal order effect assessment - 1st vs. 2nd, 3rd ,4th, reasoning blocking.
*Logistic regression; **Linear regression.
